# Injectable Micro-Hydrogel for DNA Delivery: A Promising Therapeutic Platform

**DOI:** 10.3390/jfb15030059

**Published:** 2024-03-01

**Authors:** Sunghyun Moon, Jong Bum Lee

**Affiliations:** Department of Chemical Engineering, University of Seoul, Seoul 02504, Republic of Korea; se6992@uos.ac.kr

**Keywords:** alginate ball, CpG microparticle, long-term culture, injectable hydrogel, immune stimulation

## Abstract

Utilizing the immune system as a strategy for disease prevention and treatment is promising, especially with dendritic cells (DCs) playing a central role in adaptive immune responses. The unique properties of DCs drive interest in developing materials for cell-based therapy and immune modulation. Injectable systems require syringe-compatible scaffolds, while hydrogels, like alginate, known for their programmability and biocompatibility, offer a versatile platform for immune medicine enhancement through easy preparation and room-temperature cross-linking. In this study, we synthesized alginate balls loaded with DCs or cytosine–phosphorothioate–guanine deoxyribonucleotide (CpG DNA) microparticles, aiming for long-term immune cell culture with potential immune stimulation effects. Encapsulated DCs exhibited proliferation within the alginate balls for up to 7 days, and CpG MPs were uniformly dispersed, which can facilitate uptake by DCs. This was supported by the result that DCs effectively phagocytosed CpG microparticles in a 2D environment. After the uptake of CpG MPs, the alginate balls with CpG-MP-uptaken DCs were synthesized successfully. The injectable properties of the alginate balls were easily modulated by adjusting the syringe needle gauges. This innovative strategy holds substantial promise for advancing medical treatments, offering effective and comfortable solutions for controlled immune modulation.

## 1. Introduction

In recent decades, the field of immunotherapy has emerged as a promising frontier in the battle against cancer, leveraging the body’s immune system to recognize and eliminate malignant cells. Despite notable successes, challenges persist, particularly in achieving the targeted and precise delivery of therapeutic agents to enhance efficacy while minimizing off-target effects. This challenge has led to the convergence of immunotherapy and nanotechnology, presenting an avenue for innovative solutions.

Immunotherapy proves to be a highly promising strategy for preventing and treating diseases. Dendritic cells (DCs), recognized as the most potent antigen-presenting cells within the immune system, play a central role in adaptive immune responses [1]. DCs interact with antigens at the site of foreign substance invasion, effectively stimulating T cells against the specific antigen when accompanied by pro-inflammatory signals. In the absence of such signals, peripheral tolerance to the antigen can be induced. The distinctive properties of DCs, central to the immune system, have garnered significant attention for the development of materials for cell-based therapy through immune modulation [2,3,4,5].

In the dynamic area of immunotherapy, the integration of micro-hydrogels has emerged as a transformative strategy, introducing precision and adaptability to immune modulation. Micro-hydrogels, miniature three-dimensional networks of hydrophilic polymers, prove to be versatile vehicles for controlled drug delivery and immune system modulation [6]. Characterized by their small size and high-water content, micro-hydrogels offer an innovative approach to drug delivery and immune modulation. Their unique properties, including tunable size, biocompatibility, and the capacity to encapsulate various therapeutic agents, position them as ideal candidates for precision medicine. In the context of immunotherapy, micro-hydrogels serve as intelligent carriers, facilitating the controlled release of immunomodulatory agents to orchestrate precise immune responses.

The design of micro-hydrogels enables the localized delivery of immunomodulatory agents to specific tissues or tumor microenvironments. This spatial precision enhances therapeutic efficacy by concentrating the immune-modulating payload at the intended site while minimizing systemic exposure [7]. This localized approach addresses the challenge of off-target effects often associated with conventional systemic drug administration in immunotherapy, presenting an opportunity for enhanced treatment outcomes. The modular nature of micro-hydrogels facilitates their integration into combination therapies, allowing for the simultaneous delivery of multiple therapeutic agents. This multifunctional capability is particularly advantageous in the context of immunotherapy, where the intricate interplay of various components is often necessary for optimal therapeutic outcomes. Micro-hydrogels can serve as platforms for the co-delivery of immunomodulators, antigens, and other therapeutic payloads, offering a comprehensive approach to immune system engagement.

Simultaneously, the introduction of DNA microparticles adds a novel dimension to the intersection of DNA nanotechnology and immunotherapy. Operating as versatile carriers, DNA microparticles have the ability to absorb and release therapeutic payloads in response to specific stimuli [8]. This responsiveness enables the controlled and sustained release of immunomodulatory agents, addressing the temporal dynamics of the immune response and optimizing the overall therapeutic impact. Furthermore, this property expands their applications to imaging, where nanoflowers equipped with imaging agents offer precise diagnostic capabilities. Moreover, the modifiable surfaces of DNA nanoflowers facilitate the targeted delivery of therapeutic agents, making them invaluable assets in the pursuit of personalized and precise medicine.

Hydrogels, widely employed in drug delivery, tissue engineering, and cell manipulations, stand out for their programmability and biocompatibility, making them excellent candidates to enhance the impact of immune medicine [3]. The introduction of natural polymer-based hydrogel systems represents a pivotal avenue in biomaterials research, offering versatile and biocompatible platforms with diverse applications in the fields of drug delivery, tissue engineering, and biomedical sciences. Hydrogels, composed predominantly of natural polymers such as alginate, chitosan [9], silk [10], and gelatin [11], have garnered significant attention due to their inherent biocompatibility, biofunctionality, and tunable properties. This burgeoning interest stems from the desire to develop biomimetic materials that closely mimic the physiological environment, fostering interactions with biological entities while minimizing adverse effects. Alginate, a commonly used hydrogel ingredient, is preferred due to its hydrophilicity, biocompatibility, and biodegradability [12,13]. Alginate hydrogels with various shapes can be easily prepared through crosslinking with calcium ions at room temperature [14]. Inspired by these properties, alginate hydrogels were employed as a scaffold for various applications [15,16,17,18].

This innovative hybrid approach harmonizes the precision of DNA with the biological functionality of alginate, presenting a versatile and adaptable material for a range of biomedical applications. Throughout this paper, we delve into the synergistic properties of DNA and alginate-based injectable micro-hydrogels, elucidating their potential to revolutionize immunotherapeutic effects by interaction with DCs and CpG sequences. In addition, the incorporation of therapeutic CpG DNA and alginates into micro-hydrogels facilitates DC delivery.

In this study, we focused on the synthesis of alginate micro-hydrogels loaded either with DCs or CpG-DNA-MP-uptaken DCs for cell-based immune-modulation. The primary objective was to establish a platform for long-term immune cell culture, intending to explore potential immune stimulation effects with particularized CpG DNA. The large size of CpG MPs facilitates their internalization through the phagocytic pathway. Subsequently, these DCs loaded with CpG MPs are encapsulated within size-controllable alginate balls. Importantly, our system allows for the long-term retention of DCs within the alginate balls, making it a promising platform for effective immunotherapy. This study introduces a novel approach distinct from other studies involving cell-laden microgels. Therefore, these enriched injectable micro-hydrogels hold great promise for advancing therapeutic strategies in hydrogel-based drug delivery, offering a platform for tailored and effective immunotherapy.

## 2. Materials and Methods

### 2.1. Cell Culture

DC2.4 mouse dendritic cell lines were purchased from Sigma-Aldrich (Seoul, Republic of Korea). The cells were cultured in RPMI1640 buffered with HEPES (Gibco, Grand Island, NY, USA) supplemented with 10% FBS, 1× antibiotic–antimycotic.

### 2.2. Synthesis of DC-Loaded Alginate Balls

Alginic acid (Sigma-Aldrich) was solubilized in DPBS (Gibco) at a 100 mg/mL concentration. Next, the DCs were detached and the cell numbers were counted using a hemocytometer. A total of 1 × 10^4^ cells were centrifuged and resuspended with alginic acid and transferred into a 27-gauge syringe (needle inner diameter: 0.21 mm). Finally, the alginic acid mixed with cells was dropped from a height of 1 cm into CaCl_2_ solutions prepared with a concentration of 10 mM and washed twice with DPBS.

### 2.3. Fabrication of CpG DNA Microparticles (CpG MP)

Methods of the synthesis of CpG DNA microparticles were described in detail in previous studies. Briefly, circular DNA (10 μM) were prepared by ligating phosphorylated linear DNA with T4 DNA ligases overnight at room temperature. Two circular DNAs (0.5 μM) were incubated with dNTP mix (2 mM), Phi29 polymerase (1 U μL^−1^), and reaction buffer at 30 °C for 20 h. The reactants were sonicated to break up aggregates and centrifuged at 3000× *g* for 5 min. The particles were washed twice with nuclease-free water. To label the CpG MPs with Cy5, Cy5-dCTP (0.08 mM) was added to the reaction mixture. The DNA concentration was quantified using Nanodrop.

### 2.4. Synthesis of CpG-MP-Loaded Small Alginate Balls

To synthesize CpG-MP-loaded small alginate balls, 10 μg of Cy5-labeled CpG MPs was pelleted and resuspended with 100 μL alginic acid solution (100 mg/mL). Next, the mixture of alginic acid and CpG MPs was transferred into syringes with a 31-gauge needle (needle inner diameter: 0.133 mm). Then, the mixtures were dropped from a height of 1 cm into CaCl_2_ solutions prepared with a concentration of 10 mM and washed twice with nuclease-free water.

### 2.5. Uptake Experiment

DC2.4 cells were plated in 4-well chambers at a density of 1 × 10^5^ cells per well 24 h prior to CpG MP treatment. Cells were treated with Cy5-labeled CpG MPs at a DNA concentration of 8 μg mL^−1^ for 24 h. After incubation, the cells were washed twice and fixed with formaldehyde in PBS solution for 10 min. For membrane staining, WGA-CF555 dyes were diluted 1000 times in PBS and incubated for 15 min. To stain the nucleus of the cells, Hoechst33342 dyes were diluted 10,000 times in PBS and incubated for 5 min. After staining, the cells were washed with PBS and mounted for confocal microscopy.

### 2.6. Synthesis of CpG-Uptaken DC-Loaded Alginate Balls

DC2.4 cells were plated in 4-well chambers at a density of 1 × 10^5^ cells per well 24 h prior to CpG MP treatment. Cells were treated with Cy5-labeled CpG MPs at a DNA concentration of 8 μg mL^−1^ for 24 h. After incubation, the cells were detached and the cell numbers were counted using a hemocytometer. A total of 1 × 10^4^ cells were centrifuged and resuspended with alginic acid and transferred into a 27-gauge syringe (needle inner diameter: 0.21 mm). Finally, the alginic acid mixed with CpG-uptaken DC cells was dropped from a height of 1 cm into CaCl_2_ solutions prepared with a concentration of 10 mM and washed twice with DPBS.

## 3. Results

**Figure 1:**
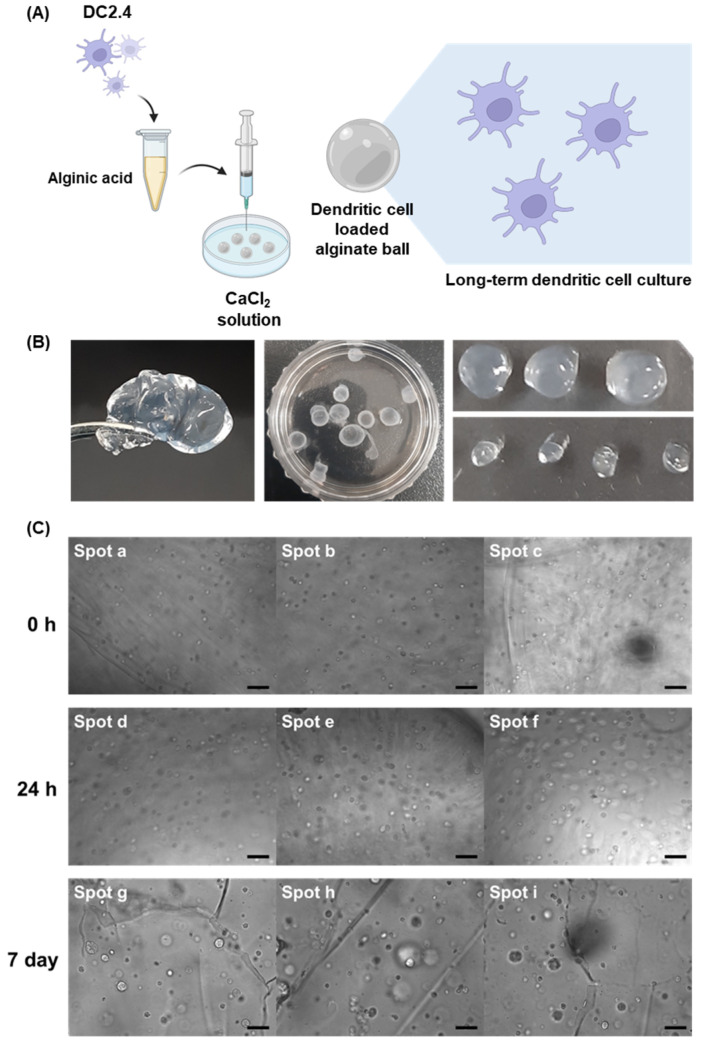
(**A**) Schematic illustration of the synthesis process of DC-loaded alginate balls, illustrating the key steps involved in encapsulating DCs within the alginate matrix (created with BioRender.com). (**B**) Digital images of alginate balls loaded with DCs. (**C**) This set of images demonstrates the viability and stability of DCs over an extended period within the alginate matrix (scale bar: 40 μm).

Micro-hydrogels can serve as platforms for the co-delivery of immunomodulators, antigens, and other therapeutic payloads, offering a comprehensive approach to immune system engagement. In this study, we focused on the synthesis of alginate micro-hydrogels loaded either with DCs or CpG-DNA-MP-uptaken DCs for cell-based immune-modulation. The primary objective was to establish a platform for long-term immune cell culture, intending to explore potential immune stimulation effects. As a proof of concept, we developed micro-hydrogels which can effectively carry cells (Figure 1A). We utilized a simple microdroplet concept by dropping gel precursors into the reaction solution.

In our pursuit of sustainable and effective DC delivery, we harnessed the capabilities of alginate gels, leveraging the unique properties of alginic acid to form gentle gels through the introduction of divalent Ca^2+^ ions. Alginate biopolymer chains were crosslinked by ionic interaction with Ca^2+^ ions which is a type of physical crosslinking. The synthesis process involved dropping alginic acid into a CaCl_2_ solution, resulting in the formation of alginate balls, as shown in Figure 1B.

These fabricated gels exhibited a distinctive spherical morphology, attributed to the alginic acid forming drops at the syringe tips. The high viscosity of the gel ensured that these drops maintained a spherical shape due to elevated surface tension. The fabricated alginate gels with 27-gauge syringes have an average diameter of 3.55 mm.

To investigate the loading efficiency of cells into the hydrogel and long-term cell proliferation, we observed the morphological changes of the encapsulated DC cells and hydrogel after 7 days. Immediate observations post synthesis, as shown in Figure 1C, revealed the presence of viable cells within the alginate balls. Notably, the number of cells within the balls increased significantly after 24 h, and these cells remained viable within the balls for an extended period of up to 7 days, which is supported by the result that the fluorescence signals of CFSE live-cell staining dyes from DCs were still observable until 7 days. This remarkable longevity suggested that alginate balls provide an environment conducive to DC survival, positioning them as a promising scaffold for sustained immune cell delivery.

**Figure 2:**
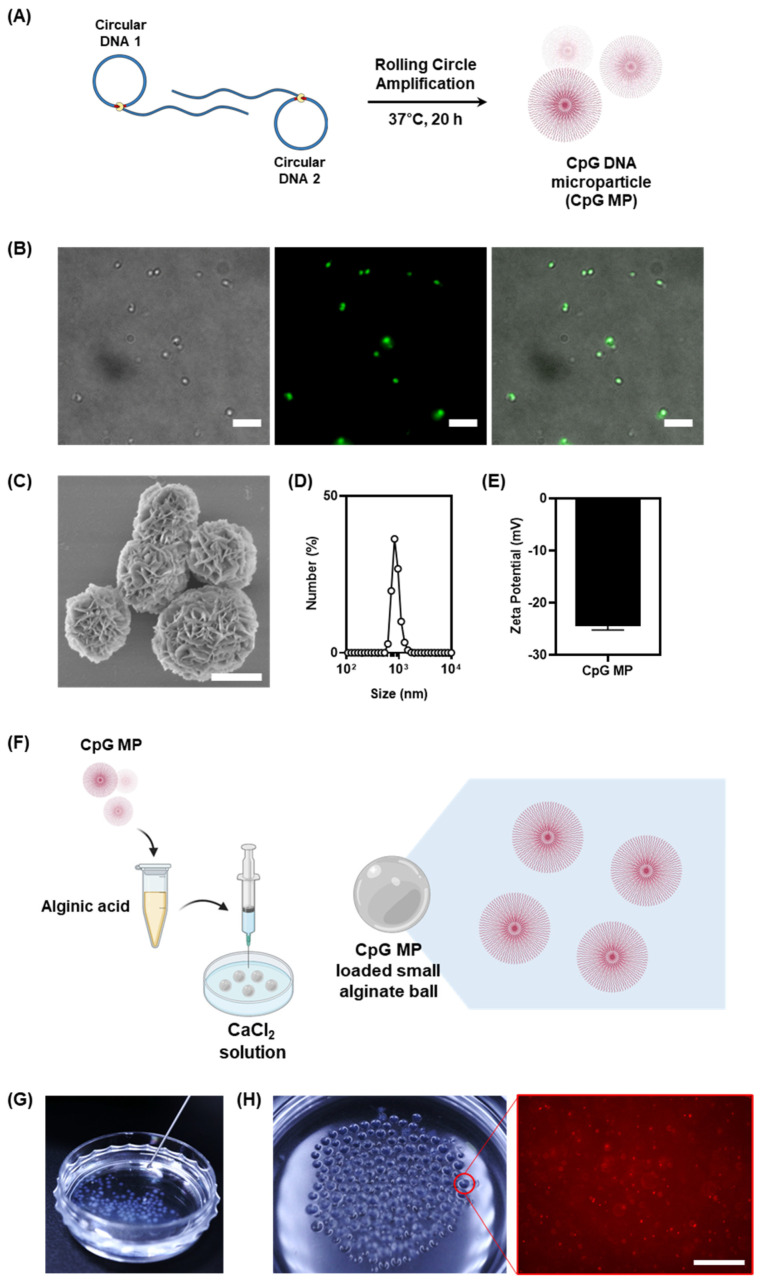
(**A**) Schematic illustration of the fabrication of CpG DNA microparticles by Rolling Circle Amplification. (**B**) Fluorescence images of the CpG MPs. The particles were stained with SYBR Green I (scale bar: 10 μm). (**C**) SEM images of the CpG MPs (scale bar: 1 μm). (**D**,**E**) The sizes and zeta-potential of the CpG MPs. Data were obtained by dynamic light scattering. (**F**) Schematic illustration of the fabrication process for small CpG-microparticle (MP)-loaded alginate balls, illustrating the key steps involved in the synthesis (created with BioRender.com). (**G**) Digital images of the injectable features of CpG-MP-loaded alginate balls. (**H**) Visualization of small CpG-MP-loaded alginate balls through digital images of Cy5-labeled CpG MPs loaded into the alginate balls and corresponding fluorescence images revealing the distribution of CpG MPs within the small alginate ball (scale bar: 20 μm).

To enhance the efficacy of micro-hydrogel-based immunotherapy, the simultaneous delivery of an adjuvant is more efficient. In particular, there is a need for a method to massively incorporate CpG as an adjuvant into micro-hydrogels. In our quest to harness DC stimulation within alginate spheres, we employed a sophisticated approach, synthesizing micron-sized CpG-containing microparticles (CpG MPs) via the Rolling Circle Amplification strategy. This method, involving continuous polymerization over a span of 20 h, yielded microparticles with unique characteristics (Figure 2A). Several analytical methods were used to confirm the formation of microparticles, including fluorescence microscopy, scanning electron microscopy (SEM), dynamic light scattering (DLS), and the zeta potential. The formation of microparticles was first confirmed by fluorescence microscopy after staining with a DNA-specific dye. Upon staining these microparticles with SYBR Green I DNA intercalating dyes, a visually striking green fluorescence was observed, underscoring the successful incorporation of CpG. To further confirm the final morphology of the CpG microparticles, the structure was confirmed by SEM (Figure 2C). Intriguingly, the microparticles exhibited intricate flower-like structures, adding an aesthetic dimension to their functional properties. It is expected that the efficiency of immune response induction will increase when immune cells are immersed in a micro-hydrogel with CpG microparticles of approximately 1 μm in size, which is easily taken up by immune cells.

To more accurately measure the size of the microparticles, they were further analyzed using DLS (Figure 2D). Accurate measurements of the microparticles revealed an average size of 1 μm, showcasing their finely tuned dimensions. The DLS size measurements were in good agreement with the fluorescent microscopic measurements. The utilization of SYBR Green I dyes not only facilitated visualization but also emphasized the precision in particle sizing. The electrostatic properties of the microparticles were further analyzed using the zeta potential to confirm that the particles were composed of DNA (Figure 2E). An essential attribute of these microparticles lies in their negative surface charge of −23 mV, attributed to the phosphate backbone of the DNA. This characteristic plays a crucial role in their interaction within the microenvironment, influencing their behavior and potential applications.

The synthesis of CpG MPs via Rolling Circle Amplification not only demonstrates a successful strategy for incorporating CpG into micron-sized particles but also highlights the versatility of these particles for potential applications in various fields. The ability to achieve DC stimulation within alginate microenvironments opens new avenues for targeted interventions, paving the way for advancements in biotechnology, medicine, and beyond. As we delve deeper into the intricacies of these innovative microparticles, the potential for groundbreaking discoveries and applications becomes increasingly evident.

Building upon this foundation, we investigated the possibility of long-term immune cell culture provided with immune-stimulant materials of our alginate-gel-based culture system by introducing CpG DNA microparticles (CpG MPs) into the gel matrix (Figure 2F). It was reported that the diameter of the syringe needle affects the volume of the droplet [19]. The facile production of smaller alginate balls with an average diameter of 0.83 mm, therefore, was readily achieved by changing syringe needles from 27-gauge to 31-gauge. This modification proved crucial for effectively encapsulating and delivering CpG MPs, transforming the alginate gel into an injectable size range (Figure 2G). The successful incorporation of CpG MPs was demonstrated by labeling them with Cy5 and examining their distribution within the synthesized small alginate balls. Our observations, as depicted in Figure 2H, showed a homogeneous dispersion of CpG MPs throughout the alginate gel. This strategic integration not only highlighted the versatility of our hydrogel system but also emphasized its potential for long-term immune cell culture with enhanced immune-stimulant properties.

**Figure 3:**
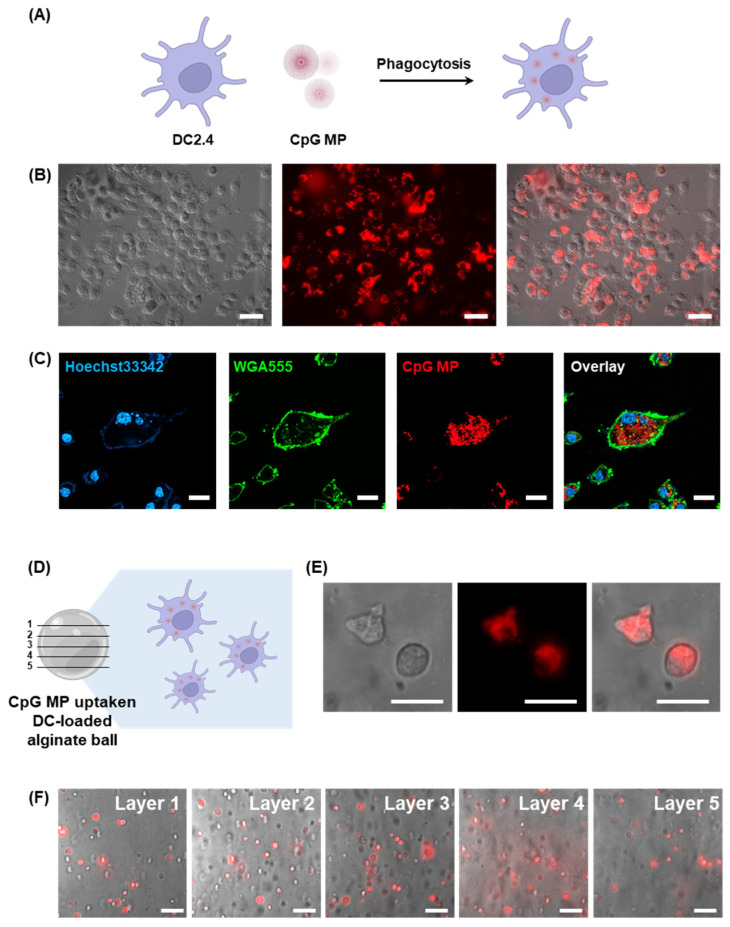
(**A**) Schematic illustration depicting the process of DCs phagocytosing CpG MPs (created with BioRender.com). (**B**) Microscopic analysis combining DIC and fluorescence images to show the uptake of CpG MPs by DCs (scale bar: 20 μm). (**C**) Confocal microscopy images providing detailed visualization of the internalization of CpG MPs by DCs (scale bar: 20 μm). (**D**) Schematic illustration of CpG-MP-uptaken DC-loaded alginate ball. (**E**) Microscopic analysis combining DIC and fluorescence images to show the loading of CpG-MP-uptaken DCs into the alginate ball (scale bar: 10 μm). (**F**) Merged images of DIC and fluorescence images of DCs inside the different layers of the alginate ball in the same spot (Scale bar: 20 μm).

To more effectively deliver the CpG-based adjuvant to increase DC cell activity, we first treated 1 μm CpG MPs, the size most easily uptaken by immune cells (Figure 3A). Further investigation focused on the encapsulation of CpG-MP-uptaken DCs into the alginate balls. To better identify intracellular uptake, Cy5-labeled rNTP was added during the RCA process and reacted with the resulting Cy5-labeled microparticles. First, we analyzed the uptake of CpG MPs by DCs, particularly DC2.4 cells known for their phagocytic nature. The brightfield and fluorescence images show that the microparticles are well internalized into the cell (Figure 3B). These cells exhibited efficient phagocytosis of micron-sized CpG particles, as evidenced by Cy5 signals originating from the CpG MPs within the cellular membrane. For more precise intracellular internalization, we used a confocal microscope to view the inside of the cells. Confocal microscope images revealed the intact spherical form of CpG MPs inside the cells, indicating the robust and effective phagocytosis of CpG MPs throughout the culture period (Figure 3C). As shown in Figure 3C, quite a few microparticles can be seen scattered throughout the cell. The high efficiency of internalization underscored the capacity of DCs to phagocytose numerous particles, affirming their pivotal role in the engulfing and internalizing CpG MPs.

To study the effects of cells that have taken up CpG-MPs, we used fluorescence microscopy. After phagocytosis of Cy5-labeled CpG MPs by DCs, we detached the DCs and loaded them into the alginate balls (Figure 3D). Then, DCs loaded in the alginate balls were cultured for 24 h. Figure 3E shows that Cy5 signals of a CpG MP from DCs are observable, which is loaded inside the alginate balls. This result shows that the DCs taking up the CpG-MPs are well distributed and encapsulated within the alginate bead. It also confirms that the cells are effectively seated inside the alginate bead without escaping. Also, we observed the five different layers of the alginate balls in the same spot, which indicates that the CpG-MP-uptaken DCs are well dispersed throughout the alginate balls (Figure 3F). These results demonstrate that a variety of cells can be entrapped in the micro-hydrogels and show promise as a novel approach for cellular immunotherapy.

## 4. Discussion

Hydrogels, extensively utilized in drug delivery, tissue engineering, and cell manipulation, are distinguished by their programmability and biocompatibility, rendering them ideal candidates for augmenting the effectiveness of immunomedicine. The advent of natural-polymer-based hydrogel systems marks a significant path in biomaterials research, providing adaptable and biocompatible platforms with manifold applications in drug delivery, tissue engineering, and biomedical sciences. Hydrogels primarily comprising natural polymers like alginate, chitosan, silk, and gelatin have garnered substantial attention owing to their intrinsic biocompatibility, biofunctionality, and adjustable properties.

This study presents a straightforward approach to synthesizing alginate micro-hydrogels loaded with DCs or CpG-MP-uptaken DCs, aiming to create a robust platform for long-term immune cell culture and sustainable immune stimulation within a delivery scaffold. The rapid reaction between alginic acid and calcium ions facilitates the efficient loading of cells, underscoring the versatility of the system. The use of syringe dropping with varying gauges offers control over the sizes of the alginate balls from 3.55 mm to 0.83 mm, allowing customization for diverse application sites of the synthesized gels. Also, the alginate balls provided a long-term survival environment for DCs up to 7 days, which can maximize the local, sustained DC functioning on the injected microenvironment.

The micron-size CpG DNA MPs have advantages for the internalization of the particles with the pathway of phagocytosis. Since DCs are classified as professional phagocytes [20], they can phagocytose and process particulate antigens or particles. Phagocytosis involves the individual engulfment of particles larger than 0.5 μm in size into a plasma-membrane-derived vesicle called a phagosome [21]. This enabled us to make CpG uptaken DCs by simply treating MPs without any transfection carrier. Moreover, a previous study suggests that CpG MPs can upregulate the immunological functions of immune cells. Upon phagocytosis, these particles trigger the release of pro-inflammatory cytokines such as TNF-α and IL-6, ultimately contributing to antigen-specific immune reactions [8].

We chose to encapsulate DCs into alginate balls after the internalization of CpG MPs for the purpose of hydrogel-based immune cell delivery. This approach will allow for a comprehensive assessment of the delivery scaffold’s ability to stimulate immune cells efficiently inside the scaffold and retain the functional immune cells in the local environment. Additionally, the use of syringes with even thinner needles can produce small injectable alginate micro-hydrogels, presenting an avenue for immune cell long-term preservation and controlled delivery. In addition, the combination of structural guidance from DNA and bioactive signaling from alginates promotes cell adhesion, migration, and differentiation. This harmonious interplay could promote tissue regeneration with our micro-hydrogel, which provides a biomimetic scaffold closely resembling the natural extracellular matrix. As a result, these enriched ECMs hold great promise for advancing therapeutic strategies in regenerative medicine, offering a platform for tailored and effective tissue repair.

## 5. Conclusions

In this study, we developed alginate micro-hydrogels that carry either dendritic cells (DCs) or DCs loaded with CpG DNA MPs for cell-based immune modulation. The main goal was to establish a platform suitable for prolonged immune cell culture, aiming to investigate potential immune stimulation effects, particularly with CpG DNA. The larger size of CpG MPs facilitates their uptake through the phagocytic pathway. Following this, the DCs loaded with CpG MPs are enclosed within alginate balls. Notably, our system allows for the extended retention of DCs within the alginate balls, presenting a promising platform for effective immunotherapy. These advancements could significantly enhance the utility of the proposed system in various biomedical applications such as immunotherapy and tissue engineering.

This innovative hybrid approach seamlessly integrates the precision of DNA with the biological functionality of alginate, offering a versatile and adaptable material suitable for a wide array of biomedical applications. Throughout this paper, we have explored the synergistic properties of DNA and alginate-based injectable micro-hydrogels, shedding light on their potential to significantly enhance immunotherapeutic efficacy through interactions with DCs and CpG sequences. Furthermore, the incorporation of therapeutic CpG DNA and alginates into micro-hydrogels facilitates the targeted delivery of DCs.

Our study specifically focused on synthesizing alginate micro-hydrogels loaded with either DCs or CpG-DNA MP-uptaken DCs for cell-based immune modulation. Our primary objective was to establish a platform for the long-term culture of immune cells to investigate the potential immunostimulatory effects of specific CpG DNA. The larger size of CpG-MPs facilitates their internalization via the phagocytic pathway. Subsequently, these CpG MP-loaded DCs are encapsulated in size-controllable alginate beads.

Significantly, our system enables the extended retention of DCs within the alginate beads, offering a promising platform for effective immunotherapy. This study introduces a novel approach distinct from others employing cell-loaded microgels. Therefore, these enriched injectable micro-hydrogels hold tremendous promise for advancing therapeutic strategies in hydrogel-based drug delivery, providing a foundation for tailored and effective immunotherapy.

## Data Availability

Data are contained within the article. The data presented in this study are available on request from the corresponding author.
